# Quercetin-Ameliorated, Multi-Walled Carbon Nanotubes-Induced Immunotoxic, Inflammatory, and Oxidative Effects in Mice

**DOI:** 10.3390/molecules27072117

**Published:** 2022-03-25

**Authors:** Amira A. Sallam, Mona M. Ahmed, Mohammed A. El-Magd, Ahmed Magdy, Heba I. Ghamry, Mohammad Y. Alshahrani, Magdy F. Abou El-Fotoh

**Affiliations:** 1 Department of Forensic Medicine & Toxicology, Faculty of Veterinary Medicine, Zagazig University, Zagazig 44519, Egypt; maaw1234@yahoo.com (A.A.S.); drmonaforensic@yahoo.com (M.M.A.); profdrmagdyfekry@yahoo.com (M.F.A.E.-F.); 2 Anatomy Department, Faculty of Veterinary Medicine, Kafrelsheikh University, Kafrelsheikh 33516, Egypt; 3 Clinical Oncology and Nuclear Medicine, Faculty of Medicine, Kafrelsheikh University, Kafrelsheikh 33516, Egypt; ahmed_magdy@med.kfs.edu.eg; 4 Department of Home Economics, College of Home Economics, King Khalid University, P.O. Box 9004, Abha 61413, Saudi Arabia; hgmry@kku.edu.sa; 5 Research Center for Advanced Materials Science (RCAMS), King Khalid University, P.O. Box 9004, Abha 61413, Saudi Arabia; moyahya@kku.edu.sa; 6 Department of Clinical Laboratory Sciences, College of Applied Medical Sciences, King Khalid University, P.O. Box 9088, Abha 61413, Saudi Arabia

**Keywords:** quercetin, multi-walled carbon nanotubes, immunotoxicity, inflammation, oxidative stress

## Abstract

The expanding uses of carbon nanotubes (CNTs) in industry and medicine have raised concerns about their toxicity on human and animal health. CNTs, including multi-walled nanotubes (MWCNTs), have been reported to induce immunotoxic, inflammatory, and oxidative effects. Quercetin is a natural flavonoid present in many vegetables and fruits and has immunomodulatory, anti-inflammatory, and antioxidant properties. Herein, we investigated the protective effects of quercetin on pristine MWCNTs-induced immunotoxicity in mice. In comparison with two doses of MWCNTs, high doses [0.5 mg/kg body weight (BW), once intraperitoneally (IP)] caused higher immunotoxic, inflammatory, and oxidative effects than low doses (0.25 mg/kg BW, once IP). Administration of quercetin (30 mg/kg BW, IP for 2 weeks) relieved these deleterious effects as evidenced by (1) reduced spleen weight, (2) increased number of total leukocytes, lymphocytes, and neutrophils, (3) elevated serum levels of IgM, IgG, and IgA, (4) decreased lipid peroxide malondialdehyde levels and increased levels of antioxidant markers reduced glutathione, superoxide dismutase, and catalase in the spleen, (5) decreased concentrations and mRNA levels of inflammatory markers tumor necrosis factor-alpha (TNFα), interleukin 1 beta (IL1ß), and IL6 in the spleen, (6) downregulated expression of immunomodulatory genes transforming growth factor-beta (TGFß), cyclooxygenase2 (COX2), and IL10, and (7) regenerative histological changes as indicated by decreased mononuclear cell infiltration, minimized degenerative changes and restored lymphocytes depletion in the spleen. These results infer that quercetin can ameliorate MWCNTs-induced immunotoxic, inflammatory, and oxidative effects.

## 1. Introduction

Carbon nanotubes (CNTs) are a type of carbon nanomaterial that form hollow tubes with either one layer (single-walled CNTs) or multi-layers [multi-walled CNTs (MWCNTs)] [1]. CNTs possess outstanding physicochemical properties and biocompatibility, making them widely used in industrial sectors and medicine [2]. They were used as carriers for the delivery of chemotherapeutics to specifically target cancer cells [3]. The bad side of CNTs on human and animal health is their toxicity on some tissues following a long exposure [4]. MWCNTs were deposited in many organs including the liver, kidney, lung, and brain after the exposure of mice to these nanomaterials [5]. Harmful effects of CNTs included cytotoxicity, genotoxicity, and carcinogenicity [5,6,7,8]. Based on animal experiments, Mitsui-7, which is a type of MWCNTs, was classified by the International Agency for Research on Cancer (IARC) as possibly carcinogenic to humans [9]. 

To date, the actual mechanism of CNTs toxicity has not been fully elucidated yet. Some researchers attributed these toxic effects to CNTs’ ability to interfere with the function of cellular components, particularly mitochondria, through the induction of oxidative stress and inflammation [8,10,11]. In vitro and in vivo toxicological studies revealed that MWCNTs had the potential to induce the production of inflammatory cytokines [12,13,14]. CNTs can pass through the blood-brain barrier and trigger the overproduction of reactive oxygen species (ROS), causing oxidative stress damage to the mitochondria of neurons [15]. Moreover, the administration of MWCNTs significantly elevated the oxidative stress markers and decreased the antioxidant markers in rat kidneys [11,16].

Regarding CNTs effects on the immune system, there are contradictory results. Some studies reported immunosuppressive effects following intravenous injection or whole-body inhalation of MWCNTs in rodents [17,18,19]. However, other reports denoted either immunostimulant potential following CNTs injection into mice [13,14,20,21] or no effects on immune cells [12,22]. For their numerous biomedical applications, it is extremely important to be aware of MWCNTs’ harmful effects on immune cells, which could be targeted by toxicity originating from xenobiotics. Therefore, there is an extreme need for a better understanding of the effect of MWCNTs on immunity [17]. 

To overcome CNTs’ harmful potential, researchers have performed surface functionalization for CNTs, with less toxic potential for the functionalized MWCNTs than pristine MWCNTs [16,23]. However, these modifications were not enough to decrease all CNTs toxicity. Therefore, it becomes an urgent demand to find suitable, effective, and safe alternatives to be used in combination with CNTs to tackle their toxicity. Quercetin (3,5,7,3′,4′-pentahydroxyflavone), a natural aglycone flavonoid present in a wide variety of fruits and vegetables, has excellent bioavailability following the consumption of quercetin-rich foods [24,25]. It has potent anti-inflammatory, antioxidant, anti-atherosclerotic, and analgesic effects [25,26]. As one of the most potent antioxidant flavonoids, quercetin exerts its effect through the inhibition of ROS production and induction of antioxidant enzyme activities [27]. For these properties, quercetin was used in the treatment of different inflammatory conditions (gout, pancreatitis, prostatitis) in addition to coronary heart disease, neurodegenerative diseases, and various types of cancer [28,29]. Quercetin can also induce immune response through the upregulation of interferon-γ (IFN-γ) and downregulation of IL4 [30]. Moreover, quercetin has a direct immunomodulatory potential on T lymphocytes [31,32]. 

An in vitro study showed that treatment with a di-ligand complex of bovine serum albumin with quercetin, and CNTs significantly decreased the cytotoxicity of CNTs on human umbilical vein endothelial cells [33]. However, little is known regarding the in vivo protective effect of quercetin on MWCNTs-induced immune toxicity. Considering the immunomodulatory, antioxidant, and anti-inflammatory effects of quercetin, we hypothesized that co-treatment with pristine MWCNTs and quercetin could ameliorate the immunotoxicity of MWCNTs. Therefore, this study was conducted to check this hypothesis.

## 2. Materials and Methods

### 2.1. Materials

Pristine (unfunctionalized) MWCNTs (>98% carbon basis) were purchased from Sigma Aldrich (St. Louis, MO, USA, CAS # 308068-56-6). Based on data provided by the manufacturer, the outer and inner diameters of MWCNTs were 10–20 and 6 to 13 nm, respectively, and the length ranged from 2.5 to 20 µm. Quercetin (yellow to yellow-green powder with purity > 94.5%) was obtained from Sigma Aldrich (CAS # 117-39-5). Dimethyl sulfoxide (DMSO, a colorless liquid polar aprotic solvent) was obtained from El-Gomhuria Co., Cairo Governorate, Egypt. The solution of quercetin and MWCNTs was prepared by dissolving in DMSO.

### 2.2. Characterization of the MWCNTs 

MWCNTs morphology was determined by a field emission scanning electron microscope (FE-SEM, LEO SUPRA 55, Carl Zeiss, Germany) and a transmission electron microscopy (TEM, LEO 912 AB electron microscope). The samples for FE-SEM analysis were prepared by taking one drop of acetone containing the dispersed MWCNTs on a silicon wafer and allowing it to dry in a vacuum oven for 30 min. For TEM, the samples were sonicated in ethanol for 10 min, and a few drops of MWCNTs suspension were spread onto a silicon substrate and allowed to evaporate at 40 °C to dry, then digital pictures were taken [34]. Zeta potential measurements were carried out using a Malvern Zetasizer Nano ZS (Malvern, UK). The measurements were performed at 25 °C in triplicate. Samples were sonicated in distilled water and appropriately diluted before measurement.

### 2.3. Experimental Design

Sixty adult male albino mice (23.34 ± 1.23 g) were obtained from the laboratory animal farm, Faculty of Science, Zagazig University. They were caged in wire-bottom galvanized metal walls under standard environmental conditions (20–24 °C and 12 h light/dark cycle). They were fed on a balanced ration ad libitum and had free access to freshwater. Mice were acclimated for 14 days before the experiment. We followed the procedures of the Institutional Animal Care and Use Committee of Zagazig University with an ethical license number of ZU-IACUC/2/F/136/2019 and were in agreement with the guidelines of the Declaration of Helsinki. 

Mice were randomly divided into 6 groups (10/group). In the first group (control, Cnt), mice were intraperitoneally (IP) injected once with 200 µL DMSO (vehicle) at the beginning of the experiment (day 1). In the second group (Que), mice were IP injected with 0.5 mL (30 mg/kg body weight) quercetin daily for 2 weeks [35]. In the third group (MWC-L), mice were IP injected once with 200 µL (0.25 mg/kg body weight) MWCNTs (low dose) at the beginning of the experiment [10]. In the fourth group (MWC-H), mice were IP injected once with 200 µL (0.5 mg/kg body weight) MWCNTs (high dose) at the beginning of the experiment. In the fifth group (MWC-L+Que), mice were IP injected once with a low dose of MWCNTs on day 1 of the experiment and IP injected with 0.5 mL (30 mg/kg body weight) quercetin daily from day 1 to day 14 of the experiment. In the sixth group (MWC-H+Que), mice were injected as in the fifth group but with a high dose of MWCNTs. 

### 2.4. Sampling

At the end of the experiment (day 14), mice were euthanized by exsanguination, and blood samples were drawn from the heart, set for 30 min, and centrifuged at 3000× *g* for 10 min at 4 °C to get serum (for serum biochemical analysis). After dissection, spleens were immediately removed, weighed, and washed with phosphate-buffered saline (PBS) to remove blood. Spleen specimens were divided into 3 parts. The first part was preserved in 10% formalin (for histopathology). The second part was kept at −80 °C (for real-time PCR). The third part was homogenized with PBS, centrifuged (10,000× *g*/15  min/4  °C), and the supernatant was kept at −20  °C (for tissue biochemical assay).

### 2.5. Biochemical Assays

Serum levels of IgG, IgM, and IgA were measured using mice ELISA Kits following the manufacturer’s protocol (MyBioSource, San Diego, CA, USA, Cat # MBS261432, MBS2510638, and MBS564073, respectively). Concentrations of TNFα (Abcam, Cambridge, UK, ab208348), IL6 (Abcam, ab222503), and IL1β (MyBioSource, Cat # MBS175967) were determined in spleen homogenates using mice ELISA Kits following the manufacturer’s guidelines.

### 2.6. Oxidant and Antioxidant Assays in the Spleen

Levels of the lipid peroxidation biomarker malondialdehyde (MDA), the concentration of reduced glutathione (GSH), and activity of antioxidant enzymes superoxide dismutase (SOD) and catalase (CAT) were measured in spleen homogenates using commercially available kits (Biodiagnostics, Giza, Egypt) following the manufacturer’s protocol and as previously described [36]. 

### 2.7. Real-Time PCR

The relative expression of the immunomodulatory and pro-inflammatory genes (TNFα, IL1ß, IL6) and immunomodulatory genes (TGFß, COX2, IL10) in the spleens of mice following treatment with MWCNTs and/or Que was evaluated by real-time PCR (qPCR). After extraction of RNA using Gene JET RNA Purification Kit (Thermo Scientific, # K0731, Walham, MA, USA), cDNA was synthesized by reverse transcription (Thermo Scientific, #EP0451) and was added to a PCR tube containing 2XMaster Mix (QuantiTect SYBR Green, Hilden, Germany ) and primers (Table 1). The tube was placed in the thermal cycler (Step One Plus, Applied Biosystem, Waltham, MA, USA) which was programmed to set the thermal cycles as follow: initial denaturation (94 °C/4 min/1 cycle), denaturation (94 °C/40 s/40 cycles), annealing (60 °C/30 s/40 cycles), and extension (72 °C/30 s/40 cycles). The expression of all candidate genes relative to the *β actin* (housekeeping) gene was presented as fold change mean ± standard error of mean (SEM) using the Livak method and as previously described [37,38,39].

### 2.8. Histopathology

Following their overnight fixation in 10% formalin, spleen specimens were histologically processed to obtain paraffin sections (5 µm). All issue slides were stained with hematoxylin and eosin and examined by a light microscope.

### 2.9. Statistical Analysis

Data were presented as mean ± standard error of mean (SEM). Significant differences among different groups were determined by One way ANOVA followed by Tukey’s Honestly Significant Difference test using GraphPad Prism 5 (GraphPad Software, Inc., LaJolla, CA, USA). Significance was declared at *p* < 0.05.

## 3. Results

### 3.1. Characterization of MWCNTs

**Figure 1:**
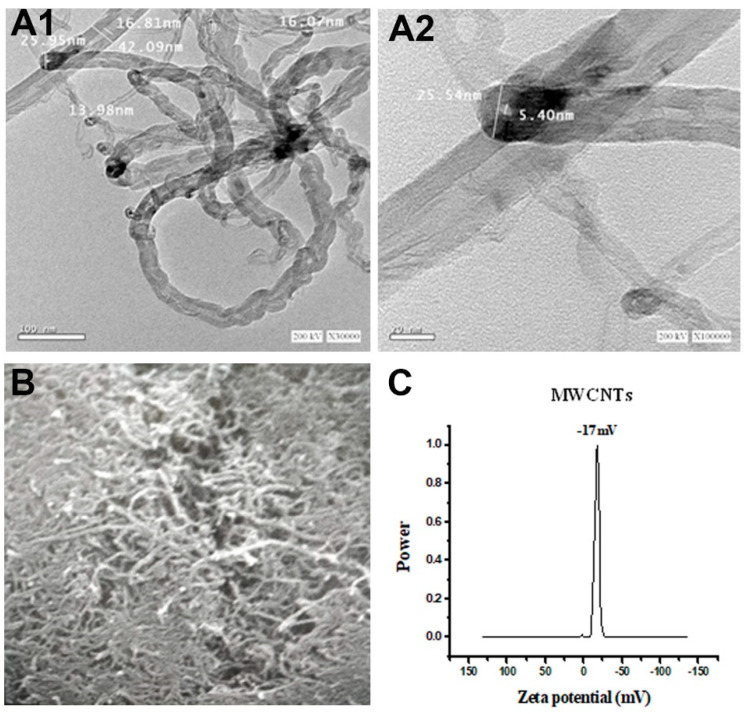
Characterization of pristine MWCNTs by transmission electron microscope (**A1**,**A2**), scanning electron microscope (**B**), and zeta potential (**C**).

Electron microscope images taken by TEM and SEM showed pristine MWCNTs in the form of hollow tubes with diameters and lengths compatible with the data given by the manufacturer (Figure 1A,B). The diameters of the MWCNTs were further confirmed by zeta potential and also were similar to the information provided by the manufacturer (Figure 1C). 

### 3.2. Effect of MWCNTs and/or Quercetin on Body and Spleen Weight

Table 2 shows the effect of MWCNTs and/or quercetin on the initial and final body weight and spleen relative and absolute weights in mice. No significant difference was noticed in the body weight of mice among all 6 groups. However, spleen absolute and relative weights were significantly increased in mice injected with MWCNTs at low (MWC-L group) and high (MWC-H group) doses, with the highest weight in MWC-H-treated mice, compared to the control (Cnt group) and quercetin-treated (Que group) mice. Mice cotreated with MWCNTs and quercetin showed significantly lower spleen absolute and relative weights, with the lowest weight in MWC-L+Que, compared to mice injected with MWCNTs-H alone.

### 3.3. Effect of MWCNTs and/or Quercetin on Total and Differential WBC Count

MWCNTs significantly declined the total WBC count, lymphocytes %, and neutrophils % relative to control and quercetin-treated groups (Table 3). However, animals cotreated with MWCNTs and quercetin showed a significantly higher WBC count, lymphocytes %, and neutrophils % than animals treated with MWCNTs alone. No significant differences in monocyte, eosinophil, and basophil% were noticed among all groups. 

### 3.4. Effect of MWCNTs and/or Quercetin on IgG, IgM, IgA Serum Levels

Mice injected with MWCNTs exhibited a significant decline in serum levels of IgG, IgM, and IgA, with the lowest levels in animals treated with high doses, compared to the control and quercetin-treated groups (Table 4). However, the co-treated groups showed a significant increase in the levels of the three Igs, with higher levels in the MWCNTs-low dose + quercetin group relative to MWCNT-treated groups. However, the enhanced Igs levels in the co-treated groups remained significantly lower than in the control and quercetin-treated groups.

### 3.5. Effect of MWCNTs and/or Quercetin on Splenic Antioxidants and Oxidative Stress Markers 

**Figure 2:**
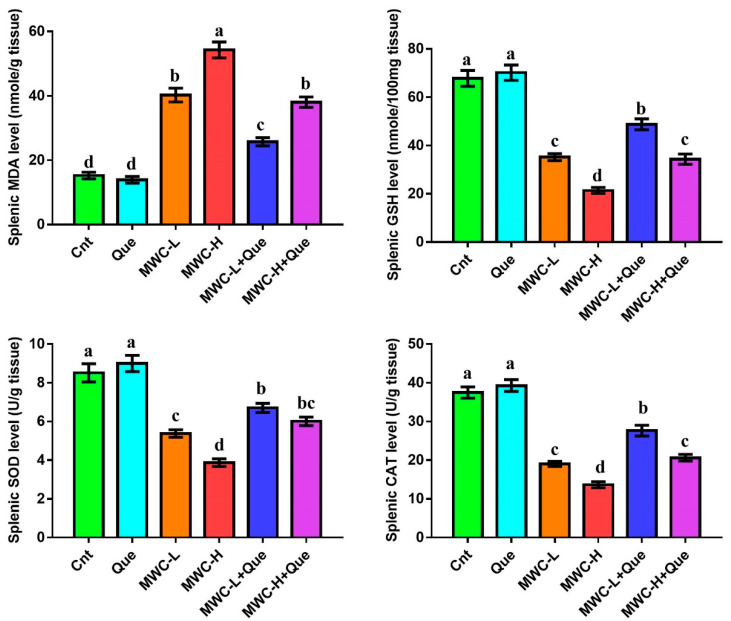
Effect of MWCNTs and/or quercetin on splenic antioxidants and oxidative stress markers. Data represent three independent experiments and were presented as mean ± SEM (n = 5/group). Means within columns carrying different letters are significantly different at *p* ≤ 0.05. All groups were compared to each other. Cnt, control group; Que, quercetin-treated group; MWC-L, MWCNTs low dose-treated group; MWC-H, MWCNTs high dose-treated group; MWC-L+Que, MWCNTs low-dose and quercetin co-treated group; MWC-H+Que, MWCNTs high-dose and quercetin co-treated group.

MWCNTs-treated mice showed significantly higher MDA and lower GSH, SOD, and CAT levels in the spleen than the control and quercetin-treated groups (Figure 2). Animals treated with higher doses of MWCNTs had higher MDA and lower antioxidant levels than mice injected with lower doses. However, mice co-treated with both MWCNTs and quercetin had significantly lower MDA and higher GSH, SOD, and CAT levels than MWCNTs-treated mice. Among the two co-treated groups, the MWCNTs-low dose + quercetin group showed the lowest levels of MDA and highest levels of antioxidant markers.

### 3.6. Effect of MWCNTs and/or Quercetin on Inflammatory Cytokines in Spleen

**Figure 3:**
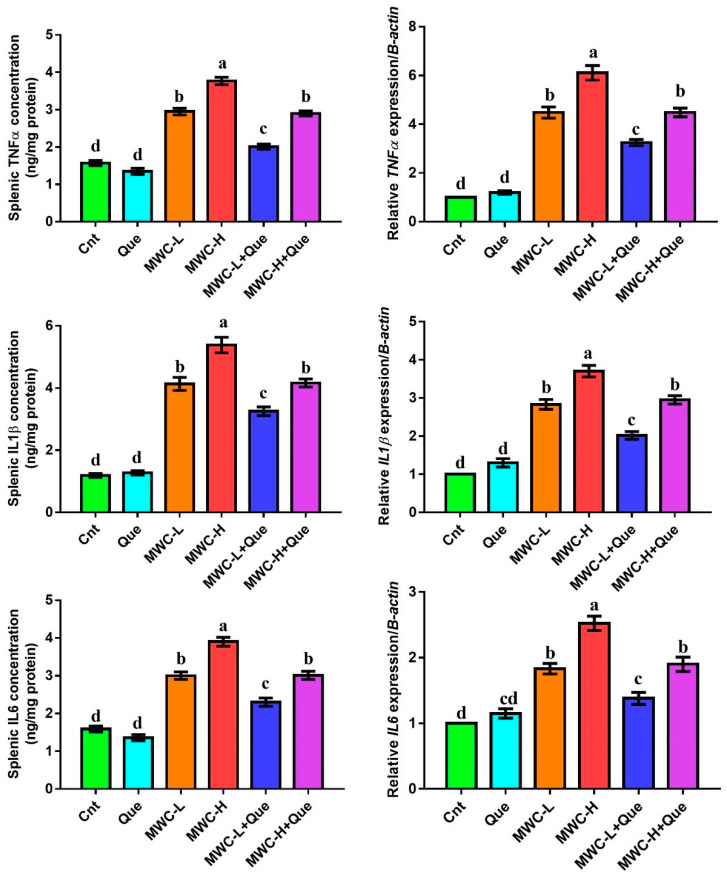
Effect of MWCNTs and/or quercetin on splenic inflammatory cytokines (TNFα, IL1β, IL6) as detected by Elisa (concentration) and qPCR (gene expression). Data represent three independent experiments and were presented as mean ± SEM (n = 5/group). Means within columns carrying different letters are significantly different at *p* ≤ 0.05. All groups were compared to each other. Cnt, control group; Que, quercetin-treated group; MWC-L, MWCNTs low dose-treated group; MWC-H, MWCNTs high dose-treated group; MWC-L+Que, MWCNTs low-dose and quercetin co-treated group; MWC-H+Que, MWCNTs high-dose and quercetin co-treated group.

Changes in concentration and expression of inflammatory cytokines (TNFα, IL1β, IL6) were detected in the spleen following the injection of MWCNTs and/or quercetin using Elisa and qPCR, respectively (Figure 3). The concentration and expression of the TNFα, IL1β, and IL6 were significantly increased in the spleens of mice injected with MWCNTs, with the highest levels in animals treated with MWCNTs high doses, compared to control and quercetin-treated mice. However, the co-administration of MWCNTs and quercetin resulted in significant decreases in the concentration and mRNA levels of these inflammatory cytokines, with the lowest levels in the MWC-L+Que group relative to the two MWCNTs groups.

### 3.7. Effect of MWCNTs and/or Quercetin on Immunomodulatory Genes in the Spleen

**Figure 4:**
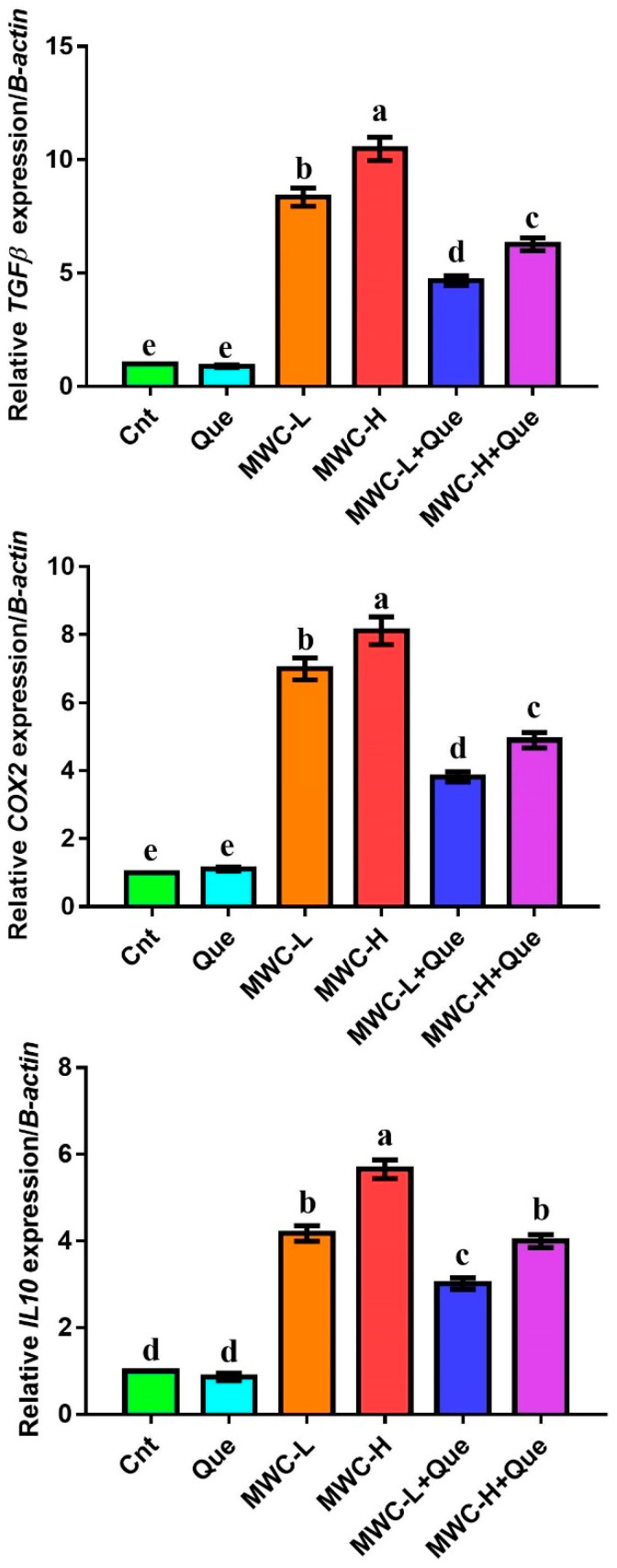
Effect of MWCNTs and/or quercetin on the relative expression of immunomodulatory genes (TGFβ, COX2, IL10) as detected by qPCR. Data represent three independent experiments and were expressed as mean fold changes ± SEM (n = 5/group). Means within columns carrying different letters are significantly different at *p* ≤ 0.05. All groups were compared to each other. Cnt, control group; Que, quercetin-treated group; MWC-L, MWCNTs low dose-treated group; MWC-H, MWCNTs high dose-treated group; MWC-L+Que, MWCNTs low-dose and quercetin co-treated group; MWC-H+Que, MWCNTs high-dose and quercetin co-treated group.

Relative expressions of immunomodulatory genes (TGFβ, COX2, IL10) were detected in the spleen by qPCR (Figure 4). Animals injected with MWCNTs exhibited significantly upregulated expressions of TGFβ, COX2, and IL10 genes, with the highest expression in MWC-H compared to the Cnt and Que groups. On the other hand, mice co-injected with MWCNTs and quercetin had a significantly downregulated expression of these immunomodulatory genes, with the lowest expression in the MWC-L+Que group compared to the two MWCNTs groups.

### 3.8. Effect of MWCNTs and/or Quercetin on Histology of Spleen

**Figure 5:**
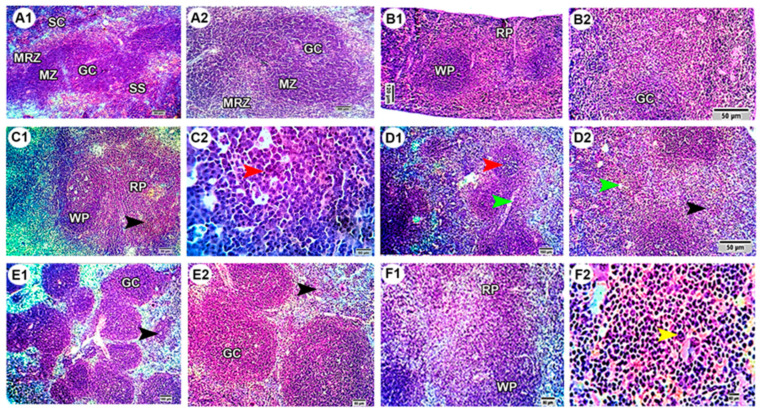
Photomicrographs of spleen sections stained with H&E. (**A**) control group, (**B**) quercetin-treated group, (**C**) MWCNTs low dose-treated group, (**D**) MWCNTs high dose-treated group, (**E**) MWCNTs low-dose and quercetin co-treated group, (**F**) MWCNTs high-dose and quercetin co-treated group. Arrowheads and labels were explained in the main text. Scale bars, 80 µm (**A1**,**A2**), 120 µm (**B1**), 50 µm (**B2**). Low (**A1**–**F1**) and high (**A2**–**F2**) magnification images were provided for each group.

Sections from the spleens of the control group revealed normal histo-morphological structures regarding the stromal framework (capsule and trabeculae) and the parenchymal structures of the white pulp including germinal centers (GC), mantle (MZ), and marginal zones (MRZ) and the red pulp involving splenic sinusoids (SS) and splenic cords (SC) (Figure 5(A1,A2)). Similarly, the spleens of mice treated with quercetin also showed normal histo-morphological structures of white pulp (WP) and red pulp (RP) with normal germinal centers (GC) (Figure 5(B1,B2)). However, the spleens of mice injected with MWCNTs in a low dose exhibited moderate histopathological alterations in the form of moderate depletion of the lymphoid elements of WP with focal apoptotic changes (red arrowhead) in the cells of GC, in addition to notable reticuloendothelial histiocytic proliferation (black arrowhead) in RP (Figure 5(C1,C2)). These histopathological changes become more evident in the spleens of mice treated with a high dose of MWCNTs, as indicated by the marked depletion of WP lymphoid cells with ill-distinct MZ and MRZ, apoptosis of GC lymphoid cells (red arrowhead), and blurred boundaries between WP and RP as well as dilated sinusoids (green arrowhead) and reticuloendothelial histiocytosis (black arrowhead) in RP (Figure 5(D1,D2)). On the other hand, notable regenerative changes were noticed in the spleens of the co-treated groups with better improvement in the MWCNTs low-dose and quercetin co-treated group, which showed normal stromal and parenchymal structures of WP, particularly in GC, with only mild to moderate lymphoid depletion besides mild reticuloendothelial histiocytosis (black arrowhead) in RP (Figure 5(E1,E2)). In the MWCNTs high-dose and quercetin co-treated group, the spleen had moderate depletion of WP lymphoid cells with ill-distinct MZ and MRZ, besides reticuloendothelial histiocytosis and megakaryocytes (yellow arrowhead) in RP (Figure 5(F1,F2)). 

## 4. Discussion

For their excellent physicochemical properties and biocompatibility, CNTs were widely used in industry and medicine. However, their extensive uses cause harmful effects and toxicities including immunotoxicity [4,17,40]. These adverse effects are mediated through, at least in part, induction of oxidative stress damage, inflammation, and immunomodulation. The majority of previous studies focused on CNTs toxicity and how they can damage cells and tissues, but there are scanty reports about the treatment or prevention potentials of drugs/agents on CNTs-induced damage. Herein we provided, for the first time, the potent immunostimulant, anti-inflammatory, and antioxidant flavonoid quercetin as a potential preventive agent that could ameliorate immunotoxicity, inflammatory, and oxidative stress induced by pristine MWCNTs in mice. Quercetin exerts this effect through the inhibition of immunomodulatory-, inflammatory-, and oxidative stress-related markers. 

In the present study, injection of pristine MWCNTs induced immunosuppressive effects as indicated by (1) the induction of splenomegaly, (2) reduced total leukocytes, lymphocytes, and neutrophils count, (3) declined serum levels of IgM, IgG, and IgA, (4) downregulated expression of immunomodulatory genes TGFß, COX2, and IL10, and (5) lymphoid depletion in the spleen. Consistent with our findings, Mitchell et al. [18,19], Zhang et al. [17], and Zhou et al. [40] have also reported immunosuppressive effects for MWCNTs in mice. The spleen is the main immune organ in the animal body, and its swelling due to inflammation interferes with its function. Subsequently, splenomegaly is a sign of immunosuppression. Our results supported by the results of Zhang et al. [17] and Zhou et al. [40] showed that the injection of MWCNTs either IP (our study) or intravenously (IV) significantly increased the spleen relative and absolute weight but with no significant changes in body weight. Similarly, Umeda et al. [41] did not find any significant changes in rat body weight after whole-body inhalation of MWCNT for 4 weeks. In contrast, IP injection of mice with phosphorylcholine-grafted MWCNTs at a higher dose of 250 mg/kg significantly increased body weight with a higher spleen relative weight [42]. This infers that the dosage of MWCNTs plays an important role in its toxicity.

The immunosuppressive effects of MWCNTs were further confirmed by reduced counts of total leukocytes, lymphocytes, and neutrophils and serum levels of IgM, IgG, and IgA. During the immune response to xenobiotics, IgM appears earlier but lasts for short time, while IgG comes later and lasts for a longer time [43]. Igs, particularly IgM, IgG, and IgA, reflect the status of humoral immunity. In agreement with our results, Zhang et al. [17] also reported significantly lowered serum levels of IgM and IgG following IV injection of 0.5 mg/kg body weight MWCNTs in mice. In support, a very recent study has also shown a significant increase in IgG and IgA in the serum of mice IV injected with 10 mg/kg body weight of MWCNTs [40]. These results suggest that MWCNTs could disturb the humoral immune response and be compatible with a reduced number of lymphocytes in these animals. In contrast, some researchers reported either immunostimulant effects for CNTs (as marked by increased lymphocytes count and serum levels of IgG and IgM) in mice [13,14,20,21] or no in vitro effects on T lymphocytes, NK cells, and monocytes [12,22].

At the molecular level, we found that IP injection of MWCNTs significantly upregulated the expression of immunomodulatory genes TGFß, COX2, and IL10 in the spleen. Similarly, whole-body inhalation of MWCNTs inhibited systemic immune response through induction of COX2 activity and upregulation of IL10 gene/protein and TGFß gene in the spleen and lung of mice [18,19]. COX2, an enzyme that induces the initial step in the production of prostanoids, is linked to inflammatory and immunosuppressive conditions [44]. The COX2/prostaglandin E2 (PGE2) signal pathway can inhibit the proliferation of NK and T lymphocytes and block T-cell IL-2 autocrine activity [45]. IL10, a known immunosuppressive cytokine, is triggered by PGE2 [18]. Moreover, TGFß also has immunoregulatory and anti-inflammatory roles through the inhibition of T cell proliferation and macrophages activation and the induction of COX2 release [46]. MWCNT inhalation could induce the release of TGFß from the lung into the spleen through circulation, and once reaching the spleen, TGFß could initiate the COX2/PGE2 pathway resulting in IL10 synthesis, ultimately inhibiting T cell proliferation and dropping immune response [18].

The balance between oxidants and antioxidants is crucial for cells to keep their normal functions [47]. If ROS exceed endogenous antioxidant enzyme activities due to inhibition of redox balance, ROS overproduce and can cause oxidative stress [48,49]. Our results revealed that spleens of mice injected with MWCNTs had significantly higher levels of the lipid peroxidation biomarker MDA but with significantly lower levels of antioxidant markers GSH, SOD, and CAT. Consistent with our findings, Zhou et al. [40] also found lower SOD activity in the spleens of mice injected IV with MWCNTs for 30 days. However, Zhou et al. [40] did not find any significant changes in ROS levels. These conflicts may be attributed to the marker used to determine the oxidative stress. We used MDA while Zhou et al. [40] used DCFH-DA. MWCNTs trigger oxidative stress damage of testis [50] and neurons [15] in mice. MWCNTs increased the oxidative stress marker MDA and decreased the antioxidant markers in the rat kidney [16]. The large surface area of CNTs triggered free radical overproduction [51]. These free radicals dominate the endogenous antioxidant enzymes leading to the damage of protein, lipids, and DNA and, finally, apoptosis [11,52]. 

In the present study, injection of MWCNTs induced inflammation in the spleen as revealed by increased concentrations and mRNA levels of inflammatory markers TNFα, IL1ß, and IL6. In support, several previous studies reported that MWCNTs induced inflammation through the upregulation of a large number of inflammatory markers including IL1α, IL6, IL12, IL13, IL17, IL3, and TNFα [12,13,14,16]. The inflammatory potential of MWCNTs was supported by histopathological changes which showed notable signs of inflammation, including congestion and mononuclear cells infiltration in the form of reticuloendothelial histiocytic proliferation in the red pulp. The overproduction of inflammatory cytokines by MWCNTs could cause mononuclear cells (mostly lymphocytes and macrophages) infiltration, which could damage the spleen. 

We found notable histopathological lesions in the spleens of MWCNTs high dose-injected mice. Spleens of these mice had ill-distinct mantle and marginal zones and blurred boundaries between white and red pulps. Similar histopathological alterations were reported by Zhou et al. [40] in mice following the IV injection of MWCNTs for 30 days. In contrast, previous studies did not find any histopathological changes in the spleen after the administration of MWCNTs in mice [18,19]. Zhang et al. [17] also did not observe notable changes in the spleen after light microscope examination, but they found pathological changes in mitochondria of splenocytes after TEM examination. Mitochondria are among the main target organelles of NPs. When NPs enter mitochondria, they induce ROS release, which negatively affects the mitochondrial membrane, potentially leading to apoptosis [11,16,53]. We also found apoptotic cells in the splenocytes of mice injected with MWCNTs. Therefore, MWCNTs could trigger apoptosis-dependent ROS and mitochondrial dysfunction in the splenocytes. As previously mentioned, the spleens of mice injected with MWCNTs showed excessive mononuclear cell infiltration of mostly macrophages and lymphocytes. Phagocytes play an important role in the transportation of CNTs from the site of the entrance, which could be lung (inhalation), intestine (ingestion), or peritoneum (injection), into circulation and subsequently to various organs [5,11,16]. MWCNTs are engulfed by macrophages, consequently triggering them to secrete IL12, which further induces T lymphocytosis [14]. This immunostimulant potential was followed by upregulated expression of TNFα and IL6 in the tissue. Similarly, we also found a higher concentration and expression of TNFα and IL6 in the spleens of MWCNTs-treated mice. We also found reticuloendothelial histiocytosis and megakaryocytes in the spleens of mice treated with MWCNTs. 

The injections of quercetin relieved all harmful effects induced by MWCNTs in mice. Animals co-treated with MWCNTs and quercetin had improved immune response as indicated by smaller spleen weight, a higher number of total leukocytes, lymphocytes, and neutrophils; higher serum levels of IgM, IgG, and IgA; decreased mRNA levels of TGFß, COX2, and IL10; and reduced lymphoid depletion in the spleen. A similar immunostimulant effect for quercetin was reported by Nair et al. [30] who found that the administration of quercetin significantly reduced pro-inflammatory cytokines including IL4. Quercetin can also directly activate T cells proliferation through the regulation of the Erk2/MAP pathway [31]. On the other hand, mice co-treated with MWCNTs and quercetin had less oxidative stress-induced damage as revealed by decreased lipid peroxide MDA levels and increased levels of antioxidant markers GSH, SOD, and CAT in the spleen. This ameliorative effect of quercetin could be due to its potent antioxidant properties, which are mediated by the inhibition of ROS release and activation of antioxidant enzyme activities [54]. Additionally, our results revealed that co-treatment with MWCNTs and quercetin significantly reduced inflammatory conditions triggered by MWCNTs, as evidenced by decreased concentrations and mRNA levels of inflammatory cytokines TNFα, IL1ß, and IL6 and minimal mononuclear cell infiltration in the spleen. These findings may be attributed to quercetin-potent, anti-inflammatory activities [55,56].

The toxic effects of MWCNTs were dose-dependent, and the MWCNTs low-dose + quercetin group showed the best improvement in immunological, antioxidant, inflammatory markers with considerable regeneration to the spleen. MWCNTs are used in the manufacturing of diagnostic medical tools and biosensors [57], and so concerns should be taken to prevent their toxic influence. As a limitation of the current study, further studies are needed to determine the actual mechanisms of action by which quercetin minimized MWCNTs’ immunotoxic, inflammatory, and oxidant effects. Considering that the physicochemical properties of pristine and functionalized MWCNTs are responsible for the different immune responses [17], it is also important to compare the protective effect of quercetin on both types of MWCNTs.

## 5. Conclusions

To the best of our knowledge, this is the first study to report that quercetin can ameliorate MWCNTs-induced immunotoxic, inflammatory, and oxidative effects. Treatment with quercetin relieved these harmful effects as indicated by higher immunological parameters/markers (increased leukocytes count, IgM, IgG, and IgA levels, and downregulated TGFß, COX2, and IL10 expression), higher antioxidant and lower oxidant markers as well as lower concentrations and mRNA levels of TNFα, IL1ß, and IL6 in the spleen. Thus, quercetin could be used as a therapeutic/preventive agent to decrease MWCNTs-induced immunotoxic, inflammatory, and oxidative effects. Further pre-clinical and clinical trials are required to validate the safety and efficacy of this novel treatment. 

## Figures and Tables

**Table 1:** Primers used for real-time PCR.

Gene	Forward Primer	Reverse Primer
TNFα	GACAAGGCTGCCCCGACTACG	CTTGGGGCAGGGGCTCTTGAC
IL1ß	AAATCTCGCAGCAGCACATCAA	CCACGGGAAAGACACAGGTAGC
IL6	TCCAGTTGCCTTCTTGGGAC	GTACTCCAGAAGACCAGAGG
TGFß	GCAACATGTGGAACTCTACCAGA	GACGTCAAAAGACAGCCACTCA
COX2	CAAGGGAGTCTGGAACATTG	ACCCAGGTCCTCGCTTATGA
IL10	CGGGAAGACAATAACTGCACCC	CGGTTAGCAGTATGTTGTCCAGC
B-actin	ACTATTGGCAACGAGCGGTT	CAGGATTCCATACCCAAGAAGGA

**Table 2:** Effect of MWCNTs and/or Que on body weight and spleen weight.

Groups	Initial Body Weight (g)	Final Body Weight (g)	Spleen Absolute Weight (mg)	Spleen Relative Weight (mg/g)
Cnt	23.28 ± 1.06	25.39 ± 1.72	82.56 ± 4.48 ^c^	3.25 ± 0.13 ^c^
Que	23.19 ± 0.89	26.26 ± 2.09	83.04 ± 5.22 ^c^	3.16 ± 0.12 ^c^
MWC-L	23.05 ± 0.93	25.47 ± 1.60	112.73 ± 5.91 ^b^	4.43 ± 0.22 ^b^
MWC-H	23.38 ± 1.15	25.08 ± 1.93	138.85 ± 7.42 ^a^	5.54 ± 0.26 ^a^
MWC-L+Que	23.65 ± 0.76	26.11 ± 2.16	104.56 ± 5.37 ^b^	4.00 ± 0.19 ^b^
MWC-H+Que	23.77 ± 0.62	25.67 ± 1.55	117.30 ± 4.68 ^b^	4.57 ± 0.20 ^b^

Data represent three independent experiments and were presented as mean ± SEM (n = 10/group). Values with different superscript letters [a (highest value)–c (lowest value)] in the same column were significantly different at *p* ≤ 0.05. All groups were compared to each other. Cnt, control group; Que, quercetin-treated group; MWC-L, MWCNTs low dose-treated group; MWC-H, MWCNTs high dose-treated group; MWC-L+Que, MWCNTs low dose and quercetin co-treated group; MWC-H+Que, MWCNTs high-dose and quercetin co-treated group.

**Table 3:** Effect of MWCNTs and/or Que on total and differential WBCs count.

Groups	WBC (10^3^/µL)	LYM (%)	NEU (%)	MON (%)	BAS (%)	EOS (%)
Cnt	5.73 ± 0.23 ^a^	76.18 ± 2.35 ^a^	22.33 ± 1.06 ^a^	1.15 ± 0.16	0.35 ± 0.03	0.22 ± 0.02
Que	5.96 ± 0.26 ^a^	77.04 ± 2.70 ^a^	21.80 ± 1.10 ^a^	1.30 ± 0.29	0.33 ± 0.03	0.25 ± 0.02
MWC-L	4.80 ± 0.21 ^b^	69.35 ± 2.03 ^b^	19.27 ± 0.69 ^ab^	0.92 ± 0.24	0.43 ± 0.04	0.18 ± 0.02
MWC-H	4.35 ± 0.22 ^b^	65.50 ± 1.96 ^b^	18.41 ± 0.83 ^b^	0.80 ± 0.20	0.49 ± 0.05	0.16 ± 0.03
MWC-L+Que	5.62 ±0.28 ^a^	74.08 ± 1.88 ^a^	20.53 ± 0.81 ^a^	1.06 ± 0.25	0.39 ± 0.04	0.20 ± 0.03
MWC-H+Que	5.36 ± 0.27 ^a^	74.57 ± 1.67 ^a^	19.68 ± 0.74 ^ab^	0.97 ± 0.28	0.44 ± 0.05	0.18 ± 0.02

Data represent three independent experiments and were presented as mean ± SEM (n = 10/group). Values with different superscript letters [a (highest value)–b (lowest value)] in the same column were significantly different at *p* ≤ 0.05. All groups were compared to each other. WBC, white blood cell; LYM, lymphocyte; NEU, neutrophil; MON, monocyte percentage; EOS%, eosinophil percentage; BAS%, basophil percentage; Cnt, control group; Que, quercetin-treated group; MWC-L, MWCNTs low dose-treated group; MWC-H, MWCNTs high dose-treated group; MWC-L+Que, MWCNTs low-dose and quercetin co-treated group; MWC-H+Que, MWCNTs high-dose and quercetin co-treated group.

**Table 4:** The effect of MWCNTs and/or Que on serum levels of IgG, IgM, and IgA.

Groups	IgG (µg/mL)	IgM (µg/mL)	IgA (µg/mL)
Cnt	1651.67 ± 70.79 ^a^	305.00 ± 12.96 ^a^	338.80 ± 11.17 ^a^
Que	1694.63 ± 67.32 ^a^	316.53 ± 10.47 ^a^	325.06 ± 10.35 ^a^
MWC-L	1378 ± 69.51 ^c^	201.67 ± 6.13 ^c^	229.00 ± 8.17 ^c^
MWC-H	1170.5 ± 58.91 ^d^	188.35 ± 7.15 ^d^	201.38 ± 7.22 ^d^
MWC-L+Que	1424.67 ±53.48 ^b^	249.33 ± 9.52 ^b^	266.63 ± 8.32 ^b^
MWC-H+Que	1345.67 ± 64.35 ^c^	213.43 ± 8.59 ^c^	231.33 ± 9.76 ^c^

Data represent three independent experiments and were presented as mean ± SEM (n = 10/group). Values with different superscript letters [a (highest value)–d (lowest value)] in the same column were significantly different at *p* ≤ 0.05. All groups were compared to each other. Cnt, control group; Que, quercetin-treated group; MWC-L, MWCNTs low dose-treated group; MWC-H, MWCNTs high dose-treated group; MWC-L+Que, MWCNTs low-dose and quercetin co-treated group; MWC-H+Que, MWCNTs high-dose and quercetin co-treated group.

## Data Availability

The data supporting the present findings are contained within the manuscript.

## Sample Availability

Samples of the compounds are not available from the authors.
